# Cysteine-10 on 17**β**-Hydroxysteroid Dehydrogenase 1 Has Stabilizing Interactions in the Cofactor Binding Region and Renders Sensitivity to Sulfhydryl Modifying Chemicals

**DOI:** 10.1155/2013/769536

**Published:** 2013-11-17

**Authors:** Lyubomir G. Nashev, Atanas G. Atanasov, Michael E. Baker, Alex Odermatt

**Affiliations:** ^1^Division of Molecular and Systems Toxicology, Department of Pharmaceutical Sciences, University of Basel, Klingelbergstraße 50, 4056 Basel, Switzerland; ^2^Department of Pharmacognosy, University of Vienna, Althanstraße 14, 1090 Vienna, Austria; ^3^Department of Medicine, 0693, University of California, 9500 Gilman Drive, La Jolla, San Diego, CA 92093, USA

## Abstract

17**β**-Hydroxysteroid dehydrogenase type 1 (17**β**-HSD1) catalyzes the conversion of estrone to the potent estrogen estradiol. 17**β**-HSD1 is highly expressed in breast and ovary tissues and represents a prognostic marker for the tumor progression and survival of patients with breast cancer and other estrogen-dependent tumors. Therefore, the enzyme is considered a promising drug target against estrogen-dependent cancers. For the development of novel inhibitors, an improved understanding of the structure-function relationships is essential. In the present study, we examined the role of a cysteine residue, Cys^10^, in the Rossmann-fold NADPH binding region, for 17**β**-HSD1 function and tested the sensitivity towards sulfhydryl modifying chemicals. 3D structure modeling revealed important interactions of Cys^10^ with residues involved in the stabilization of amino acids of the NADPH binding pocket. Analysis of enzyme activity revealed that 17**β**-HSD1 was irreversibly inhibited by the sulfhydryl modifying agents N-ethylmaleimide (NEM) and dithiocarbamates. Preincubation with increasing concentrations of NADPH protected 17**β**-HSD1 from inhibition by these chemicals. Cys^10^Ser mutant 17**β**-HSD1 was partially protected from inhibition by NEM and dithiocarbamates, emphasizing the importance of Cys^10^ in the cofactor binding region. Substitution of Cys^10^ with serine resulted in a decreased protein half-life, without significantly altering kinetic properties. Despite the fact that Cys^10^ on 17**β**-HSD1 seems to have limited potential as a target for new enzyme inhibitors, the present study provides new insight into the structure-function relationships of this enzyme.

## 1. Introduction

17*β*-Hydroxysteroid dehydrogenase (17*β*-HSD) enzymes are involved in the interconversion of inactive and active sex-steroid hormones, thereby playing an essential role in the intracrine regulation of estrogen-, androgen-, and progesterone-dependent physiological functions [1]. 17*β*-HSD1 is responsible for the conversion of estrone to estradiol, the most potent natural estrogen. The highest expression of this enzyme is found in breast and ovarian tissue. Importantly, 17*β*-HSD1 expression showed a negative correlation with breast cancer progression and was identified as an independent prognostic marker for the disease-free and overall survival of patients with breast cancer [2, 3]. Elevated expression of 17*β*-HSD1 was observed in endometrial cancer [4] and nonsmall cell lung cancer [5]. Furthermore, the expression ratio of 17*β*-HSD1 and 17*β*-HSD2 was found to be a predictor of the response to tamoxifen in postmenopausal breast cancer patients [6]. 

The administration of specific 17*β*-HSD1 inhibitors led to significantly decreased tumor growth in breast cancer cell xenograft tumor mouse models [7, 8] and reversed estrogen-dependent endometrial hyperplasia in transgenic mice [9], indicating that this enzyme is a promising drug target against estrogen-dependent diseases such as endometriosis and endometrial cancer as well as breast and ovarian tumors. Several classes of chemicals inhibiting 17*β*-HSD1 were tested *in vitro*, including steroid-like molecules, nonsteroidal compounds, and chimeric molecules, acting on both the active center and the cofactor binding site of the enzyme [8, 10–16]. However, only a limited number of inhibitors have been tested *in vivo* so far and further research is needed. In order to develop potent and selective 17*β*-HSD1 inhibitors, a profound understanding of the structure-function relationships of the enzyme is essential. 

17*β*-HSD1 belongs to the short-chain dehydrogenase/reductase (SDR) family and contains the conserved Rossmann-fold for nucleotide binding, the catalytic triad with residues Ser^142^, Tyr^155^, and Lys^159^ [17] and a dimerization region. Lys^149^ is a critical residue for the discrimination between C-18 and C-19 steroid substrates [18]. 

Targeting of functionally essential cysteine residues in proteins is suggested as a promising approach for the design of new types of pharmaceutical agents [19]. 17*β*-HSD1 contains six cysteines. In a previous study, we demonstrated that the structurally related enzyme 11*β*-hydroxysteroid dehydrogenase type 2 (11*β*-HSD2) contains a cysteine residue at position 90, analogous to Cys^10^ on 17*β*-HSD1 [20]. Mutation of Cys^90^ on 11*β*-HSD2 led to almost complete inactivation of the enzyme. Therefore, we hypothesized that Cys^10^ on 17*β*-HSD1 may represent a target site for novel inhibitors. In the present study, we applied 3D structure modeling and enzyme activity measurements to investigate the biochemical properties of Cys^10^Ser mutant 17*β*-HSD1 and to compare its sensitivity towards sulfhydryl modifying agents with that of wild-type 17*β*-HSD1. 

## 2. Materials and Methods

Cell culture media were purchased from Invitrogen (Carlsbad, CA) and [2,4,6,7-^3^H]-estrone from Amersham Pharmacia (Piscataway, NJ, USA). All other chemicals were from Fluka AG (Buchs, Switzerland) and of the highest grade available.

### 2.1. Construction of Expression Plasmids and Site-Directed Mutagenesis

In order to facilitate detection, an octahistidine tag was added to the C-terminus of human 17*β*-HSD1 by PCR amplification using oligonucleotide primers containing the tag-coding sequence (forward primer 5′-TAAACCCTGAGGAGGTGGCGGAGGTCTTC-3′, reverse primer 5′-TGCTCTAGAAGCTTAATGATGATGATGATGATGATGATGCTGCGGGGCGGCCGGAGGATCG-3′). The activity of the tagged 17*β*-HSD1 was indistinguishable from that of the untagged enzyme. The substitution of Cys^10^ to serine was introduced into the C-terminal histidine-tagged 17*β*-HSD1 cDNA in Bluescript vector by site-directed mutagenesis using the forward primer 5′-TCATCACCGGCTCTTCCTCG-3′ and the reverse primer 5′-GAGGAAGAGCCGGTGATGAG-3′ according to the Quick Change mutagenesis kit (Stratagene, Amsterdam, The Netherlands), followed by subsequent recloning into pcDNA3.1 expression plasmid. 

### 2.2. Cell Culture, Transfection, and Western Blotting

HEK-293 cells were grown to 60–70% confluence in Dulbecco's Modified Eagles Medium (DMEM) supplemented with 10% fetal bovine serum, 4.5 g/L glucose, 50 units/mL penicillin/streptomycin, and 2 mM glutamine, followed by transfection using the calcium phosphate precipitation method. The transfection efficiency was 32 ± 3%. To examine the expression levels of the overexpressed proteins, we performed SDS-PAGE (30 *μ*g of total cellular proteins loaded per lane) followed by immunoblotting and detection of the histidine-tagged proteins using tetra-His antibody (Qiagen GmbH, Hilden, Germany; Cat. No. 11561526). As a loading control *β*-Actin was visualized using an anti-*β*-actin antibody (Santa Cruz Biotechnology, Santa Cruz, CA; Cat. No. B1204).

### 2.3.  17*β*-HSD1 Activity Measurements

To determine 17*β*-HSD1 activity, HEK-293 cells were transfected by the calcium phosphate precipitation method. Cells were harvested 48 h after transfection, washed twice with phosphate-buffered saline (PBS), and centrifuged for 4 min at 150 ×g. Supernatants were removed and the cell pellets were quick-frozen in a dry ice-ethanol bath and stored at −80°C. The reductase activity of 17*β*-HSD1 was measured by incubation of cell lysates for 10 min at 37°C in the presence of 200 nM radiolabeled estrone and 400 *μ*M NADPH in a reaction buffer containing 20% glycerol, 1 mM EDTA, and 50 mM potassium phosphate, pH 7.4. Inhibitors were diluted from stock solutions in dimethylsulfoxide (DMSO) and immediately used in the assays. The DMSO concentration did not exceed 0.1% and had no effect on the enzyme activities. 

For the enzyme activity assay in intact cells, HEK-293 cells were grown in 10 cm culture dishes, transfected with plasmids for histidine-tagged wild-type 17*β*-HSD1 or Cys^10^Ser mutant, detached 24 h after transfection and distributed in 96-well plates at a density of 10,000 cells per well. After 16 h, cells were incubated in serum- and steroid-free medium, and the conversion of radiolabeled estrone to estradiol was determined upon incubation for 30 min at 37°C in a total volume of 50 *μ*L containing 200 nM estrone. The reaction was stopped by adding methanol containing 2 mM unlabeled estrone and estradiol, followed by separation of steroids by TLC and scintillation counting.

For determination of enzyme kinetics, estrone concentrations ranging from 10 to 800 nM were used. Data (mean ± SD) were obtained from three independent experiments and were calculated using the Data Analysis Toolbox (Elsevier MDL, Allschwil, Switzerland). 

### 2.4. Analysis of the Protein Stability of Wild-Type 17*β*-HSD1 and Mutant Cys^10^Ser

The stability of wild-type and mutant 17*β*-HSD1 protein was analyzed basically as described previously [21]. Briefly, to investigate the protein half-life of wild-type 17*β*-HSD1 and mutant Cys^10^Ser, HEK-293 cells grown in six-well plates were transfected with plasmids for mutant and wild-type enzyme, washed once with PBS 24 h after transfection, and incubated with fresh medium containing 50 *μ*g/mL cycloheximide. At 0, 12, 24, and 48 h, aliquots of cells were harvested, followed by immunodetection using tetra-His antibody. Longer incubations with cycloheximide were inappropriate due to toxic effects in HEK-293 cells.

Alternatively, cells transfected with plasmids for mutant and wild-type enzyme were washed once 16 h after transfection, followed by incubation in leucine-free DMEM (MP Biomedicals, Illkirch, France) for 45 min to deplete endogenous leucine. The medium was then replaced by 1 mL of leucine-free DMEM supplemented with 20 *μ*Ci/mL L-leucine-[3,4,5-^3^H(N)], followed by incubation for 3 h. The labeling was terminated by addition of 5 mM unlabeled leucine, washing twice with DMEM, and incubation in DMEM. At 0, 12, 24, and 48 h, aliquots of cells were snap-frozen prior to purification of histidine-tagged proteins by using a Ni-NTA agarose kit according to the manufacturer (Qiagen AG, Hombrechtikon, Switzerland). After elution, proteins were subjected to SDS-PAGE, the gels were dried and exposed to tritium-sensitive screens (TR uncoated BaFBr:Eu^2+^ screens) for 16 h, followed by analysis using a Cyclone Phosphor-Imager (PerkinElmer Life and Analytical Sciences, Shelton, CT, USA). 

### 2.5. Multiple Sequence Alignment

The protein sequences of 17*β*-HSD1 from different species and different human SDRs were compared using the ClustalW algorithm, run on http://www.ebi.ac.uk/, with default program parameters. 

### 2.6. Structural Modeling

To investigate the effect on the structure of 17*β*-HSD1 of mutating Cys^10^ to Ser, we extracted 1FDT from the Protein Data Bank (PDB). 1FDT contains human 17*β*-HSD1 cocrystalized with estradiol and NADP^+^ [22]. Cys^10^ was converted to serine using the Biopolymer option in the Insight II software package. The structure of the Ser^10^ mutant 17*β*-HSD1 was refined with Discover 3 for 10,000 iterations, using a distant dependent dielectric constant of 2.

## 3. Results

### 3.1. Multiple Sequence Alignments and 3D Structure Modeling

**Figure 1:**
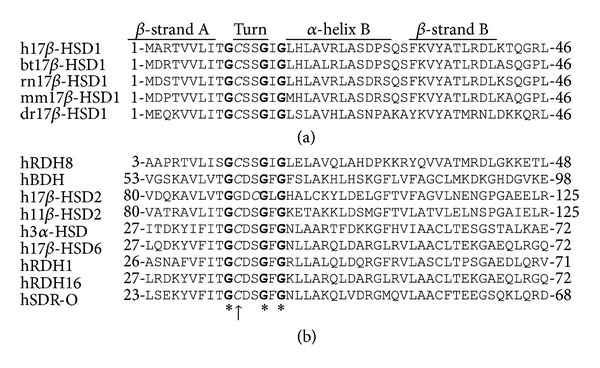
Sequence alignment of the cofactor binding domain of 17*β*-HSD1. Peptide sequences of human (h), bovine (bt), rat (rn), mouse (mm), and zebrafish (Danio rerio, dr) 17*β*-HSD1 (SDR28C1) are shown in the *upper panel* and those of the related human SDR enzymes all-trans retinol dehydrogenase RDH8 (SDR28C2), hydroxybutyrate dehydrogenase BDH (SDR9C1), 17*β*-HSD2 (SDR9C2), 11*β*-HSD2 (SDR9C3), 3*α*-HSD (SDR9C4), 11-cis retinol dehydrogenase (SDR9C5), 17*β*-HSD6 (SDR9C6), retinol dehydrogenase SDR-O (SDR9C7), and retinol dehydrogenase RDH16 (SDR9C8) in the *lower panel*. The locations of *β*-strand A through *β*-strand B are indicated above the alignment. The position of the conserved Cys^10^ on 17*β*-HSD1 is indicated by an *arrow* and the conserved glycine residues of the Rossmann-fold by asterisks (*in bold*).

The analysis of the peptide sequence of human 17*β*-HSD1 using the Clustal W algorithm [23] revealed that residue Cys^10^ is highly conserved among species, including rodents and zebrafish (Figure 1), suggesting a role for this residue in the stability and/or function of the protein. Cys^10^ is located between the first two of three highly conserved glycine residues of the Rossmann-fold nucleotide binding domain. Interestingly, the cysteine and the two downstream serine residues also are present in all-trans retinol dehydrogenase RDH8, which like 17*β*-HSD1 belongs to the SDR28C family. Moreover, the cysteine residue at this position is conserved in all members of the SDR9C family, except for 17*β*-HSD2, which has a cysteine residue downstream by two positions. 

**Figure 2:**
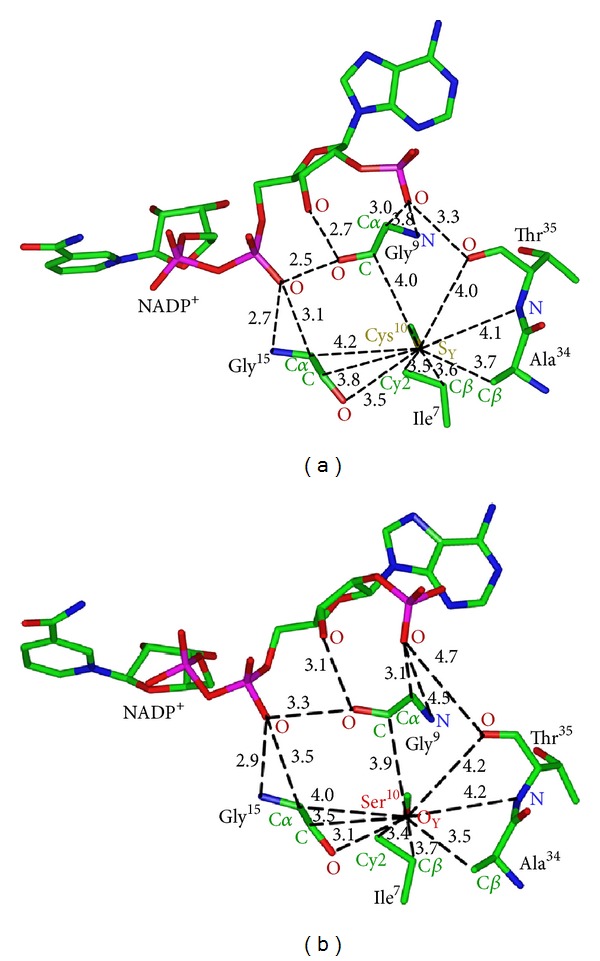
Position of Cys^10^ in wild-type 17*β*-HSD1 and of Ser^10^ in mutant 17*β*-HSD1. Cys^10^ has stabilizing interactions with neighboring residues involved directly in the binding of cofactor NADPH. *A*, wild-type 17*β*-HSD1. Cys^10^ has van der Waals contacts with Ile^7^, Gly^9^, Gly^15^, Ala^34^, and Thr^35^. Gly^9^, Gly^15^, and Thr^35^ contact NADP^+^. *B*, Cys^10^Ser mutant 17*β*-HSD1. Ser^10^ has van der Waals contacts with Ile^7^, Gly^9^, Gly^15^, Ala^34^, and Thr^35^. The backbone nitrogen on Gly^9^ and the backbone oxygen on Thr^35^ no longer contact the oxygen on the adenosine phosphate.

To begin to understand the role of Cys^10^ for 17*β*-HSD1 function, we analyzed the interactions of Cys^10^ with adjacent amino acids in 17*β*-HSD1 cocrystalized with estradiol and NADP^+^ [22]. The crystal structure of 1FDT reveals that Cys^10^ has van der Waals contacts with Ile^7^, Gly^9^, Gly^15^, Ala^34^, and Thr^35^. Gly^9^, Gly^15^, and Thr^35^ directly stabilize the binding of the cofactor NADP^+^ (Figure 2(a)). The numerous close contacts of Cys^10^ with amino acids in the NADP(H)-binding site indicate that substitution of the sulfhydryl group with a larger chemical would disrupt this site and probably alter NADP(H) binding and catalytic activity.

To begin to study the function of this cysteine residue, we substituted Cys^10^ with serine, the biochemically most similar amino acid. As revealed by the 3D model (Figure 2(b)), mutating Cys^10^ to serine does not lead to significant changes in contacts between Ser^10^ and Ile^7^, Gly^9^ and Ala^34^. However, all three contacts between Ser^10^ and Gly^15^ are shorter than between Cys^10^ and Gly^15^, and Gly^15^ is more distant from a phosphate oxygen on NADP^+^ (Figure 2(b)). Also, Ser^10^ is a little more distant from the backbone oxygen on Thr^35^, and Thr^35^ is $4.7\;\overset{´}{Å}$ from the oxygen on the adenosine phosphate. The backbone nitrogen on Gly^9^ is $4.5\;\overset{´}{Å}$ from the same phosphate oxygen on NADP^+^ (Figure 2(b)). 

### 3.2. Inhibition of 17*β*-HSD1 by Sulfhydryl Modifying Agents

In a previous study, sulfhydryl modifying chemicals were found to exert potent inhibitory effects on 11*β*-HSD2 and substitution of Cys^90^ by serine abolished enzymatic activity [20]. To test the hypothesis that Cys^10^ on 17*β*-HSD1 has similar essential stabilizing interactions in the NADPH binding region, we assessed the inhibitory potential of the cysteine modifying agent N-ethylmaleimide (NEM) and of dithiocarbamate chemicals. NEM appeared to be a weak inhibitor with an IC_50_ of 22 ± 6 *μ*M upon simultaneous incubation of lysates of 17*β*-HSD1 expressing HEK-293 cells with 200 nM estrone and increasing concentrations of NEM. In line with an irreversible mode of inhibition, preincubation with NEM resulted in a more pronounced inhibitory effect in a time-dependent manner. Preincubation of lysates with 20 *μ*M NEM for 1 h prior to the addition of estrone completely abolished 17*β*-HSD1 activity (data not shown).

Next, we tested dithiocarbamate chemicals for inhibition of 17*β*-HSD1. In contrast to the previously observed potent inhibition of 11*β*-HSD2, dithiocarbamates turned out to have rather modest inhibitory effects on 17*β*-HSD1. The IC_50_ values for thiram (21 ± 4 *μ*M), disulfiram (8 ± 1 *μ*M), maneb (25 ± 3 *μ*M), and zineb (24 ± 3 *μ*M) upon simultaneous incubation of the 17*β*-HSD1 enzyme preparation with substrate and inhibitor were about two orders of magnitude higher than those obtained for 11*β*-HSD2, indicating that 17*β*-HSD1 is much less prone to inhibition by sulfhydryl modification. 

**Figure 3:**
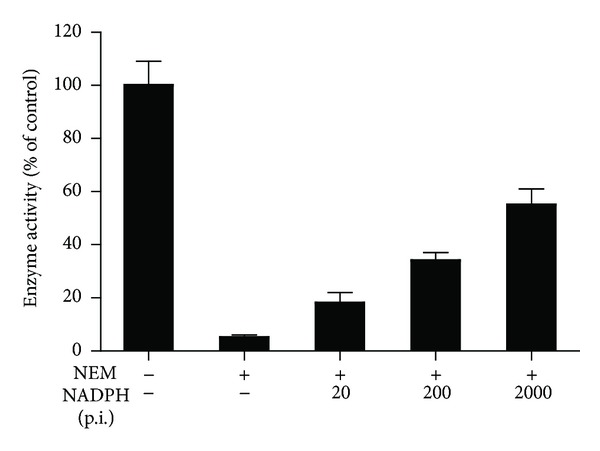
Preincubation with NADPH protects 17*β*-HSD1 from inhibition by NEM. Lysates of HEK-293 cells expressing 17*β*-HSD1 were preincubated for 15 min with saline or various concentrations of NADPH as indicated, prior to the addition of estrone (200 nM), NADPH (400 *μ*M), and the sulfhydryl modifying agent N-ethylmaleimide (NEM, 60 *μ*M). The estradiol produced was determined after 10 min of incubation. Data (mean ± SD) were normalized to the control in the absence of NEM and were obtained from at least three independent experiments measured in triplicate.

Importantly, as shown in Figure 3, preincubation with NADPH for 15 min protected from inhibition by NEM, an effect which was concentration dependent. Preincubation with NADPH also protected from inhibition by dithiocarbamates (data not shown), suggesting that binding of NADPH prevents the covalent modification of Cys^10^ in the cofactor binding region.

### 3.3. Substitution of Cys^10^ with Serine Leads to Decreased Protein Stability

**Figure 4:**
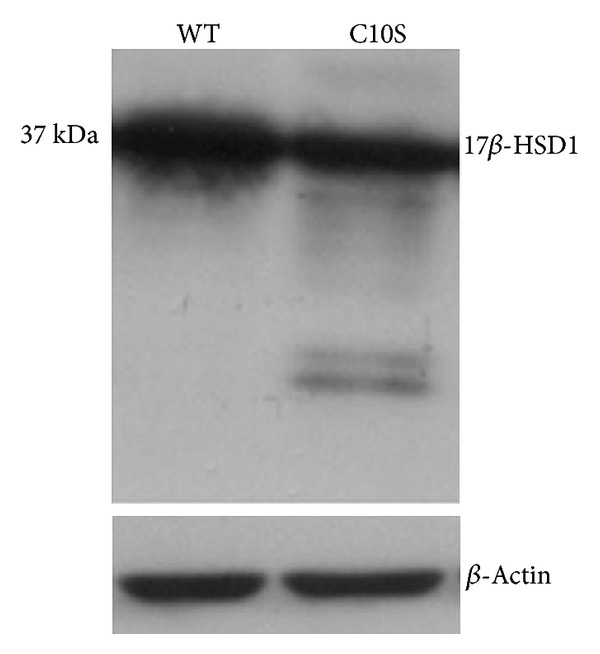
Western blot analysis of the protein expression of histidine-tagged wild-type 17*β*-HSD1 and mutant Cys^10^Ser. A representative experiment from four independent transfections is shown.

To study the role of Cys^10^ for 17*β*-HSD1 function, we generated mutant Cys^10^Ser and compared the expression of histidine-tagged wild-type and mutant enzymes upon transient expression in HEK-293 cells, which lack endogenous 17*β*-HSD1 expression. Despite comparable transfection efficiency, mutant Cys^10^Ser was expressed at approximately twofold lower levels than wild-type 17*β*-HSD1 (Figure 4). Moreover, several low-molecular weight bands were detected, indicating a lower stability of the Cys^10^Ser mutant protein.

**Figure 5:**
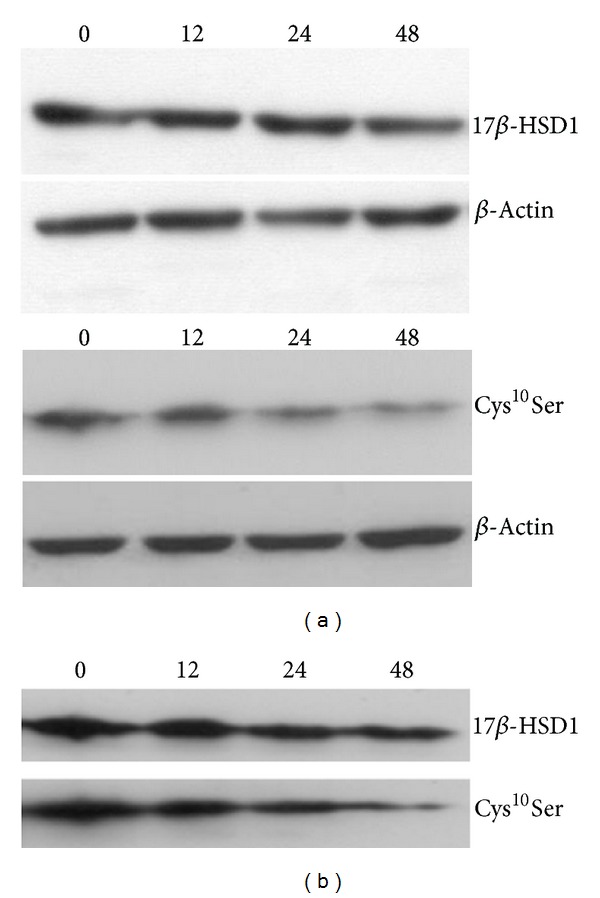
Stability of 17*β*-HSD1 and Cys^10^Ser mutant. C-terminal histidine-tagged wild-type 17*β*-HSD1 and Cys^10^Ser mutant enzymes were expressed in HEK-293 cells. *A*, 24 h posttransfection cells were incubated with 50 *μ*g/mL cycloheximide to inhibit *de novo* protein synthesis. After 0, 12, 24, and 48 h cells were harvested and the amount of expressed protein was analyzed semiquantitatively using anti-histidine antibody. *β*-actin served as control. *B*, the protein half-life of 17*β*-HSD1 and Cys^10^Ser mutant was estimated by pulse-chase experiments, labeling cellular proteins with tritiated L-leucine, followed by washing and incubation in leucine-free medium for different times. After purification of histidine-tagged proteins by Ni-NTA agarose, proteins were separated by SDS-PAGE and gels were dried and exposed to tritium-sensitive screens for detection of radioactivity by phosphor-imaging. Representative experiments are shown.

Next, we performed experiments in transfected HEK-293 cells using cycloheximide in order to block *de novo* protein synthesis and estimate protein half-life. The signal detected for histidine-tagged 17*β*-HSD1 protein was not significantly decreased 48 h after blocking translation with cycloheximide (Figure 5(a)), indicating a protein half-life greater than 48 h. Longer incubations are not appropriate due to the cytotoxicity of cycloheximide. In contrast, mutant Cys^10^Ser was less stable and the protein half-life of the mutant enzyme was estimated to be 26 ± 7 h (mean ± SD). A pulse-chase experiment using ^3^H-leucine labeling confirmed the decreased stability of the mutant protein (estimated protein half-life of 15 ± 6 h) compared with the wild-type 17*β*-HSD1 (estimated protein half-life of 36 ± 10 h) (Figure 5(b)). The differences in the protein half-life estimation by the pulse-chase and cycloheximide methods may be explained by the incomplete inhibition of protein synthesis in the latter approach. 

### 3.4. Mutant Cys^10^Ser Retains Catalytic Activity and Is Protected from Inhibition by Sulfhydryl Modifying Agents

A comparison of the enzyme activities of lysates expressing wild-type 17*β*-HSD1 or mutant Cys^10^Ser showed approximately 50% lower activity for the mutant (data not shown). However, after correction for protein expression using anti-histidine-tag antibody there was no significant difference between wild-type and mutant enzymes. The analysis of enzyme kinetics revealed a slightly higher apparent *K*_*m*_ but no significant change in the maximal velocity (*V*_max⁡_) for estrone reduction (Table 1). In intact cells, the activity of mutant Cys^10^Ser was indistinguishable from that of the wild-type enzyme (data not shown). 

**Figure 6:**
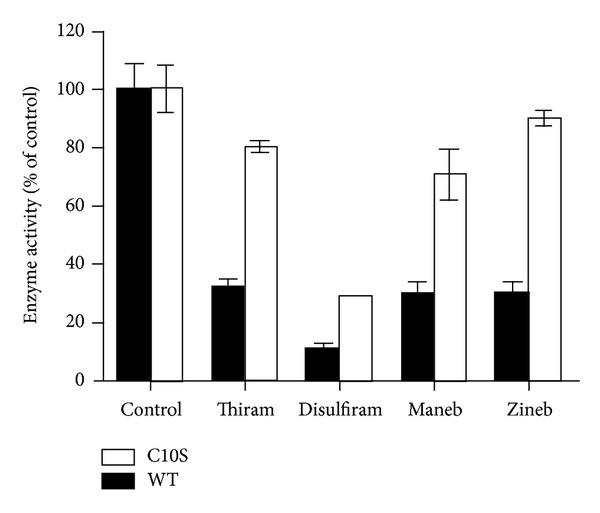
Inhibition of 17*β*-HSD1 and Cys^10^Ser mutant by dithiocarbamate chemicals. The conversion of estrone (200 nM) to estradiol in the presence of 400 *μ*M NADPH and various dithiocarbamates (80 *μ*M final concentration) was measured in lysates of transfected HEK-293 cells. Black bars, wild-type 17*β*-HSD1 enzyme (WT); white bars, Cys^10^Ser mutant 17*β*-HSD1 (C10S). The amount of total protein in the reaction was equalized to exclude differences due to unspecific binding of the inhibitors to cellular proteins.

Next, we compared the effect of different dithiocarbamate chemicals on the activity of wild-type 17*β*-HSD1 and mutant Cys^10^Ser. Mutant Cys^10^Ser was protected from inhibition by all of the dithiocarbamates tested (Figure 6). Similarly, the cysteine-modifying agent NEM inhibited the activity of the wild-type enzyme approximately two times stronger than that of mutant Cys^10^Ser, confirming the role of this cysteine for 17*β*-HSD1 function (data not shown).

## 4. Discussion

Analysis of the 17*β*-HSD1 protein sequences from different species revealed that the cysteine at position 10 in the human enzyme is highly conserved. Protein sequence alignment further revealed that a cysteine at this position in the Rossmann-fold nucleotide binding region is conserved in the SDR subfamilies SDR28C and SDR9C. The 3D structure of 17*β*-HSD1 predicts several van der Waals contacts between Cys^10^ and residues involved directly in the binding of NADPH. The 3D model of Ser^10^ mutant 17*β*-HSD1 finds that although most contacts are conserved there are changes that may be significant. For example, Ser^10^ is closer to Gly^15^, and Gly^15^ is more distant from a phosphate oxygen on NADP^+^. Also, in the Ser^10^ mutant 17*β*-HSD1, the backbone oxygen on Thr^35^ and backbone nitrogen on Gly^9^ move to 4.7 $\overset{´}{Å}$ and 4.5 $\overset{´}{Å}$, respectively, from the oxygen on the adenosine phosphate. In wild-type 17*β*-HSD1, the backbone oxygen on Thr^35^ and backbone nitrogen on Gly^9^ are 3.3 $\overset{´}{Å}$ and 3.8 $\overset{´}{Å}$, respectively, from this oxygen on NADP^+^. The loss of these two stabilizing contacts could reduce the affinity for NADP(H) in the Ser^10^ mutant 17*β*-HSD1. Another possible contribution to lower stability of NADP(H) in the Ser^10^ mutant may be the different chemical properties of the thiol group on Cys^10^ and alcohol on Ser^10^. For instance, only the thiol group is predicted to be partially deprotonated at neutral pH, which could better stabilize the site around Cys^10^. *K*_*m*_ values for NADPH will need to be determined in follow-on studies to address this question.

Nevertheless, our prediction is supported by results from Huang et al. who demonstrated that insertion of a positively charged lysine residue in the neighborhood of Cys^10^ and Ser^12^ led to a more than 20-fold increase in the preference of 17*β*-HSD1 for NADP(H) against NAD(H) [24]. In a previous study, we found that the related SDR enzyme 11*β*-HSD2 also contains a cysteine residue at the position corresponding to Cys^10^ on 17*β*-HSD1 in the cofactor binding region [20]. The 11*β*-HSD2 Cys^90^Ser mutant almost completely lost its enzymatic activity, due to impaired protein folding and mislocalization of the mutant protein. 

Interestingly, mutations of the analogous residue Cys^69^ in the human (R)-3-hydroxybutyrate dehydrogenase led to a slight increase in the apparent *K*_*m*_ for both NADP(H) and NAD(H) [25]. The Cys^69^Ser mutant of the (R)-3-hydroxybutyrate dehydrogenase, analogous to 17*β*-HSD1 Cys^10^Ser, showed a twofold lower apparent *V*_max⁡_ compared with the wild-type enzyme. The slight increase in the apparent *K*_*m*_ of 17*β*-HSD1 mutant Cys^10^Ser for estrone, observed in our measurements in cell lysates, indicates a decreased affinity for the substrate as a result of disturbed interactions with the cofactor NADPH. A careful structural comparison between the interactions of residues with the cofactor and substrate in 17*β*-HSD1, 11*β*-HSD2, and (R)-3-hydroxybutyrate dehydrogenase should offer an explanation for the different effects of the modification of the analogous cysteine residues of the three SDRs. In human retinol dehydrogenase, the function of the analogous Cys^38^ is not studied in detail; however, Boerman and Napoli showed that the protein contains cysteine residues in close proximity, which are essential for the catalytic activity [26].

In our experiments, the preincubation with increasing concentrations of NADPH was able to protect 17*β*-HSD1 from inhibition by the sulfhydryl modifying agents NEM and dithiocarbamates, suggesting an indirect role of Cys^10^ in stabilizing the binding of the cofactor.

Dithiocarbamates and NEM were able to inhibit the activity of 17*β*-HSD1, although at concentrations significantly higher than those needed for inhibition of 11*β*-HSD2. The high sensitivity of 11*β*-HSD2 toward sulfhydryl modifying chemicals was recently shown to be dependent on the presence of a cysteine residue in the substrate binding region [27]. An analogous cysteine residue is absent in the substrate binding region of 17*β*-HSD1. Our western blot experiments detected low-molecular bands for the 17*β*-HSD1 Cys^10^Ser mutant, suggesting increased degradation of the mutant enzyme. The more rapid degradation is likely due to changes in the protein conformation and thus exposure of amino acid residues normally buried inside the protein, followed by activation of the proteasome. A limitation of the present study includes that wild-type and mutant enzymes were overexpressed and expression levels are higher than endogenous levels. Thus, the estimated half-life of the proteins may be different in an endogenous situation. Nevertheless, the results demonstrate a reduced stability of the mutant compared with the wild-type enzyme. 

The present work provides novel information on the structure-activity relationship of 17*β*-HSD1 and reveals that Cys^10^ is involved in essential stabilizing interactions in the cofactor binding region. Furthermore, we showed that Cys^10^ is the target for sulfhydryl modifying agents. Future studies using protease digestion of purified 17*β*-HSD1 wild-type and Cys^10^Ser mutant proteins should provide further mechanistic insight into the stabilizing interactions of this residue. 

## Figures and Tables

**Table 1:** Enzyme kinetics of wild-type 17*β*-HSD1 and Cys^10^Ser mutant. The reduction of various concentrations of estrone (10 to 800 nM) to estradiol was measured in cell lysates from transfected HEK-293 cells in the presence of 400 *μ*M NADPH. The data were normalized according to protein expression and represent mean ± SD from four independent experiments measured in triplicate.

	*V*_max⁡_ (nmol × h^−1^ × mg^−1^)	*K*_*m*_ (nM)
17*β*-HSD1 WT	110 ± 5	43 ± 6
17*β*-HSD1 Cys^10^Ser	124 ± 3	65 ± 3
